# Group Mentorship Model to Enhance the Efficiency and Productivity of PhD Research Training in Sub-Saharan Africa

**DOI:** 10.29024/aogh.25

**Published:** 2018-04-30

**Authors:** Yukari C. Manabe, Harriet Nambooze, Elialilia S. Okello, Moses R. Kamya, Elly T. Katabira, Isaac Ssinabulya, Mark Kaddumukasa, Yvonne Nabunnya, Robert C. Bollinger, Nelson K. Sewankambo

**Affiliations:** 1 Infectious Diseases Institute, Makerere University College of Health Sciences, Kampala, UG; 2 Division of Infectious Diseases, Department of Medicine, Johns Hopkins University School of Medicine, Baltimore, Maryland, US; 3 MEPI-MESAU Secretariat, Makerere University College of Health Sciences, UG; 4 Department of Psychiatry, School of Medicine, Makerere University College of Health Sciences, Kampala, UG; 5 Department of Medicine, School of Medicine, Makerere University College of Health Sciences, Kampala, UG

## Abstract

**Introduction:**

High quality PhD training in sub-Saharan Africa is important to strengthen research evidence to advance development and health. Training a critical mass of independent investigators capable of original scientific research requires strong mentorship, research environments, and international networks. We sought to iteratively improve a PhD training model in Uganda through systems capacity building.

**Methods:**

PhD students were selected through a rigorous competitive application and selection process, which included a written proposal and a face-to-face panel interview. The program provided administrative support, paid tuition fees, tools (space, equipment, research money), skills (short research courses on study design, biostatistics, manuscript and grant writing), and infrastructure (finance, grants management support, and lab infrastructure). Guidance to identify local and international mentorship was also provided in addition to two to three group meetings per year where data was presented and progress assessed by the program leaders in addition to available local mentors.

**Results:**

Seventeen PhD students were selected, and fifteen will complete training through the MEPI-MESAU program. To date, 60% have completed, including 2 students who started 2 years into the program. So far, 169 publications have been published in the peer-reviewed literature. Our PhD students have supervised and mentored 65 Master’s students, which illustrates the cascade effect of PhD training on the academic medical school environment.

**Conclusions:**

The systems capacity building approach to PhD training is an efficient and productive training model that allowed strong outputs at lower cost and with relatively few additional mentors to rapidly achieve a critical mass of independent scientists able to conduct original research and mentor others.

## Background

It is important to strengthen research for development and health [1,2], as well as to address the barriers to achieving universal health coverage [3]. High-quality PhD training is an important part of research capacity building in low-income countries (LICs) and seeks to create a critical mass of able scientists who can perform independent, original scientific research and mentor others. In addition, prioritizing research with local relevance that can influence policy and practice should also be emphasized. PhD training is time consuming and requires supervision and mentorship, strong research networks, and exposure to strong research environments [4,5,6,7]. The opportunity cost for all involved is not trivial. Therefore, establishing high-quality training approaches is imperative.

In order to build sustainable research capacity strengthening in LICs, a recent approach increased funding to academic universities or to establish regional or national centers of research excellence often linked to these academic centers. Training of individuals is most effective within a resourced context [8]. The Medical Education Partnership Initiative (MEPI) is an innovative, ambitious, and potentially transformative five-year program established by the US Office of the Global AIDS Coordinator, the Fogarty International Center of the National Institutes of Health, and the Health Resources Service Administration (HRSA) to increase the number of well-trained doctors in Africa [9]. The original goal of MEPI was to increase the number of doctors particularly in the area of HIV/AIDS to meet critical human resource needs in sub-Saharan African countries where poverty, illiteracy, negative cultural practices, and political instability synergize to increase disparities in health care access and quality both within and between nations [10]. MEPI-MESAU (Medical Education for Equitable Services for All Ugandans) is a five-school consortium in Uganda. Within MEPI MESAU, both Johns Hopkins University and Case Western Reserve University were international partners in the consortium in the area of research capacity building.

We sought to expand in-country PhD training for students from all of the MESAU institutions based on a previously published model of PhD training [4]. The limited pool of local senior faculty members already holding PhDs who could serve as effective supervisors was a clear bottleneck. We hypothesized that focused, intensive group mentorship can supplement traditional PhD supervision and be a potentially efficient training model to build high quality research critical mass with relatively little additional human resources.

## Methods

### PhD training program description

PhD training applicants submitted a written application, including a short proposal on their proposed area of study. Based on these applications, a proportion were selected for interviews on the basis of the quality of the concept, the topic, research accomplishments as per the curriculum vitae, research potential, and letters of recommendation. Because funding was provided from the Office of the Global AIDS Coordinator, proposals that focused on HIV were encouraged. The trainee selection process was competitive and involved solicitation for candidates. The application selection process used a standardized scoring rubric and faculty consensus on candidate selection. Final recipients were selected by a multidisciplinary group comprised of at least one senior faculty member from all five Ugandan medical institutions and Johns Hopkins University, as well as the principal investigator of the MEPI-MESAU program. The selected trainees had local mentors who were selected by the students based on their specialty interest and expertise. The overarching MEPI-MESAU program also offered a chance to involve international supervisors as well, which exposed some trainees to more diverse supervision and teaching.

**Figure 1:**
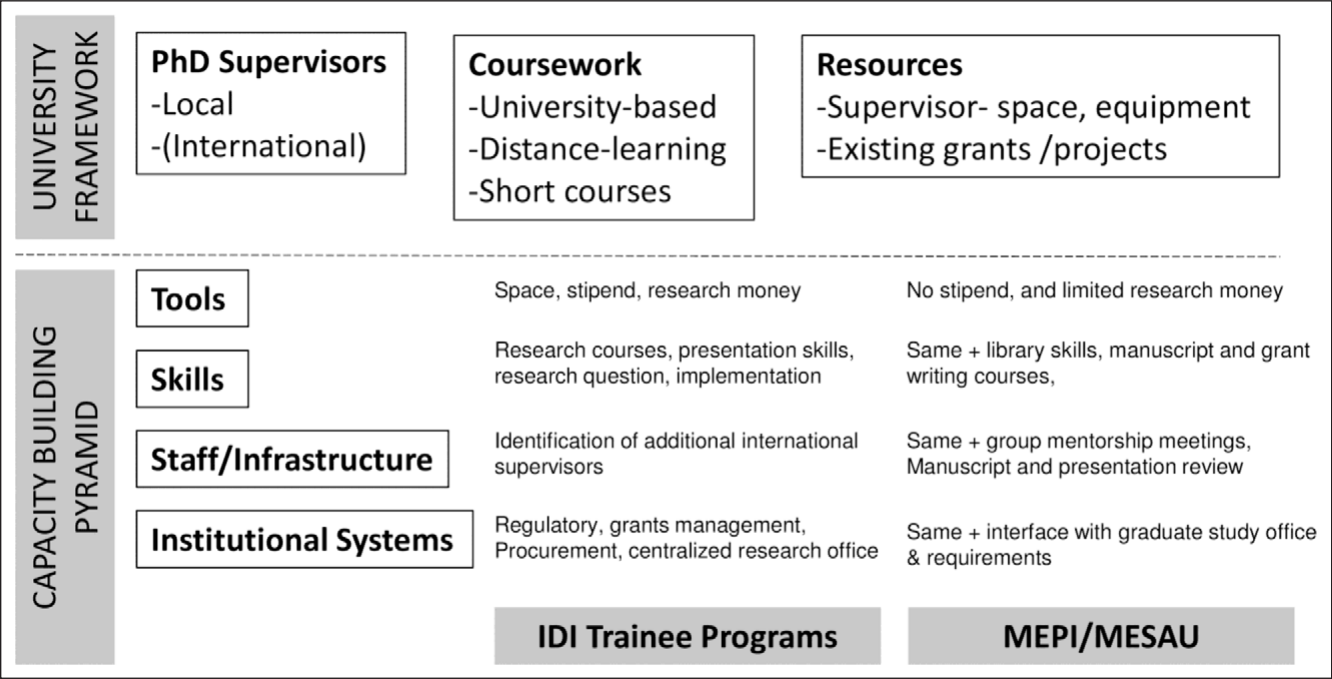
The organization of the MEPI-MESAU PhD Training Program. **Legend:** Research capacity building pyramid showing the university framework on which the program was layered. The first column shows the various levels of the pyramid. The second column shows the inputs at each level of the pyramid in the original Infectious Diseases Institute (IDI), Makerere University PhD program [4], and the third column shows the inputs in the present PhD program in MEPI-MESAU.

The central secretariat at Makerere University, College of Health Sciences (MakCHS) provided oversight of the PhD progress. The PhD coordinator arranged and moderated monthly meetings. In these meetings, the PhD coordinator guided the fellows on strategies to navigate the institutional PhD requirements and to critique different sections of draft manuscripts. In some instances, experts were invited to talk to fellows about various issues impacting their PhD studies. Trainees were required to submit progress reports every six months, signed by their supervisors with comments on progress to both the program and to fulfill University requirements. These forms were designed previously to track academic progress and outputs, as well as to ensure that supervisors remained engaged. Productivity was assessed by looking at the number of Master’s students supervised, number of peer-reviewed articles published, and number of oral or poster presentations made to scientific conferences. The underlying organization of the program was based on a previously developed model of PhD training [4]. This capacity building model is based on the Potter-Brough [11] pyramid where the upper levels offer tools (space, equipment, research money) and skills (research courses, implementation strategies), but infrastructure and attention to systems capacity building is needed for sustainability (Figure 1). The overarching MEPI-MESAU program addressed finance, grants management, and lab infrastructure as in the previous Infectious Diseases Institute model [4]. In this iteration, we offered significantly less monetary support for stipend and protected time and less research money than the previous PhD training model in order to invest more at the lower levels of the pyramid. Administrative support and program mentorship was similar across programs.

The program provided administrative support for PhD students to navigate the often complicated university higher degrees’ committee requirements and also paid all fees related to registering for a PhD training. A limited budget for research was also provided, which was particularly important for those with projects nested within larger international collaborative research projects to ensure that they could develop data in a scientifically distinct area that they could lead and first-author. The program also paid publication fees and biostatistical software fees and offered skills-building workshops, including manuscript writing, statistical analysis, grant writing, time management, short scientific research presentations, personal development planning, qualitative and quantitative research methods, and a course in using evidence to inform policy and practice (knowledge translation), as well as other university courses, such as philosophy of research. Supervisors were also offered a MESAU-funded mentorship one-week course. Only one of the students was a full time PhD student. Protected research time was variable; some trainees had block study leave for up to three years, others had only a few months at a time, and some continued to carry a heavy teaching and clinical service load due to limited faculty in their departments.

In addition to the named local, and in some cases international, PhD supervisors, the program adopted a group mentorship model due to the large number of simultaneous trainees in the program. In year one, there were two group meetings and then intensive individual meetings with each fellow to help with proposal development. Subsequently, there were two to three additional group meetings per year. Group meetings were held in a ‘lab meeting’ style: each fellow presented a focused part of their PhD work for ten to fifteen minutes to teach them to succinctly present one aspect of their study with data. Additional individual meetings for those fellows who were falling behind were suggested. A few fellows sought out distance mentorship via email and Skype for additional review of manuscripts, theses, and final presentations.

For a PhD to be awarded at a Ugandan University, students’ theses must be reviewed by an external examiner and internal examiners in written form, and students must undergo a three-hour public defense with an examination committee, in addition to an opponent who is often from an outside, internationally recognized university or research institution.

Our study utilized data that had been collected as part of ongoing monitoring and evaluation of the PhD training program supported by the grants indicated below in acknowledgments. Ethical approval for the study was obtained from the Makerere University School of Medicine Research and Ethics Committee.

## Results

Sixteen applicants were short-listed and interviewed. Eleven were selected at the end of 2011, with at least one PhD student from three 3 of the 5 consortium universities. In subsequent years, 6 additional PhDs were selected who were co-funded either through parent studies or the MEPI cardiovascular and neurology linked awards at MakCHS. Overall, 6 (35%) were women, 8 (47%) had co-supervisors outside of Uganda, 5 (29%) PhD projects were clinical trials, and 5 (29%) of the doctoral candidates had thesis projects nested within another research study. Nine (53%) thesis projects had a lab biologic assay or translational lab component. Currently, 9 (60%) students have successfully been awarded PhDs, with median time to completion 4.2 years. One (6%) other is are awaiting an oral defense date, 2 (12%) PhD students withdrew from the doctoral training, and 5 (29%) have not yet completed (4 of whom were in the late-starting group).

## Productivity

**Figure 2:**
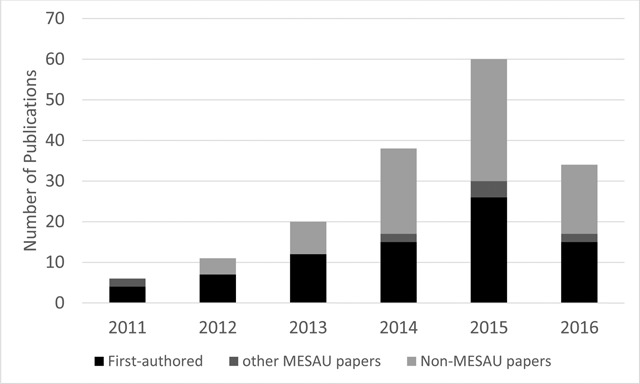
Authorship by MEPI-MESAU PhD students. **Legend:** Publications authored by MESAU PhD students. Publications are separated by First-authored by PhD student (black), Other MESAU papers (dark grey), and Non-MESAU papers published in the same period (grey).

In the published peer-reviewed literature, 89 publications acknowledge MESAU funding and are authored (first-author and co-author) by a MESAU PhD student (Figure 2). Of those, 88.9% (79 of 89) were first-authored by the MESAU student. There are an additional 80 publications where MESAU fellows are co-authors that were not directly related to the PhD thesis work. The number of publications per student ranged widely (1–24) over the PhD period.

**Figure 3:**
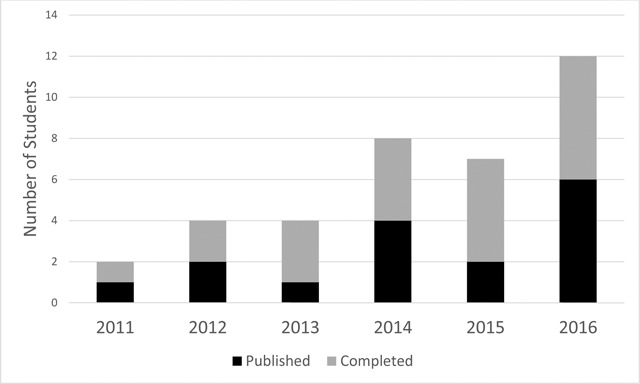
Master’s students co-mentored by PhD students and their publication outputs. **Legend:** Master’s students co-mentored by MESAU PhD students. Students are separated by those that have published their Master’s work (black) and those that have completed but have not yet published (grey).

The PhD fellows have supervised and co-mentored 65 Master’s students from September 2011 to September 2016 (Figure 3). Of these, 16 have both completed and published their thesis work, and 21 of the Master’s students have completed but not yet published their findings.

Six of the PhD students have pursued research that has led to independent grant funding as well as collaborative grants on which they are listed as a co-investigator.

Of the 15 students who remained in the program, the average total cost per student for both stipend and research costs was $55,200 (range $15,200–$99,950) over 2–4.5 years of training. One student was initially funded from another program and was supported by MESAU to complete his PhD training in the last 2 years. In comparison, the previous IDI PhD program paid yearly stipends to protect research time in addition to research costs; the average cost per student was $240,000 (range $170,000–$275,000).

Several other qualitative observations were made. Faculty supervisors were also invited to the group mentorship meetings every 4–6 months, which led to improved faculty feedback over time and greater involvement in student supervision. Second, given the emphasis placed on the biannual critiques from the program leadership and faculty supervisors, trainees in the program began independent self-organized meetings more regularly to prepare for the critiques and also to solicit the opinion and advice of their peers.

## Discussion

The PhD training program resulted in high completion rates of PhDs with independent original research, the capability to mentor others, and significant peer-reviewed publications outputs over an average of 4.2 years, which is shorter than the 5–6 year Makerere University norms. Compared to our previously presented model of PhD training, which also led to important outputs including first-author publications, sponsored research funding, and research that impacts policy and practice [4], the per student investment was much lower in this program in terms of oversight time and money to provide protected time for research and writing. In this iterative model, we continued to build sustainable capacity and were able to scale up the program through group mentorship and economies of scale. We were able to accomplish similar outcomes in terms of productivity in comparison to our previously published productivity at the IDI (2008–2011), where most of the first-authored publications were from the PhD students [4]. The approach in the new model also overcame the issue in many sub-Saharan African institutions where the absolute number of qualified PhD supervisors is too low for the number of PhD students that are needed to reach a critical mass of productive, independent scientists. The supervisors named on the PhD committees are generally overburdened with competing priorities. Interestingly, six of our primary supervisors were promoted into leadership positions, including departmental headship, over the time period of the PhD program, illustrating that the same limited number of core faculty with PhDs are tasked with administrative leadership in addition to trainee supervision.

Several unexpected outcomes were also observed. As a result of these structured meetings and the substantive scientific review that they received from senior faculty, the PhD students started small group mock presentations among themselves to critically appraise each other’s work prior to the scheduled meetings. Peer mentorship emerged as a result of the biannual critiques and was particularly effective at MakCHS where there is a critical mass of PhD trainees. Third, MESAU PhDs improved their own supervision of Master’s students, with a high proportion of their Master’s students completing a publication.

It is difficult to disentangle the relative value of any one intervention. A recent African-led initiative—the Initiative to Strengthen Health Research Capacity in Africa (ISHReCA)—identified key requirements to strengthening health-research capacity in Africa [12,13]. Our project focused on supporting the individual PhD students. Other efforts have improved the research environment by supporting the larger institution with strategic development planning, infrastructure, and promoting networks and partnerships, particularly south-south, for example between Ugandan university medical schools and also between African MEPI institutions [13]. Some of the success of this program may be due to timing; lower levels of the capacity building pyramid were also being addressed and strengthened through MEPI–MESAU and other funding, which allowed for more effective research capacity building [11].

In summary, the MESAU PhD program was able to train concurrently 15 PhD students through a program that was embedded and resourced through the larger MEPI grant. The group and peer mentorship model proved to be a mutually beneficial relationship that can build research capacity at the local institution and facilitate sustainability. The model was an efficient and productive training model, which allowed strong outputs at lower cost and with relatively few additional mentors to rapidly achieve a critical mass of independent scientists able to conduct original research and mentor others.
